# Validation of the Burden Index of Caregivers (BIC), a multidimensional short care burden scale from Japan

**DOI:** 10.1186/1477-7525-4-52

**Published:** 2006-08-18

**Authors:** Mitsunori Miyashita, Aki Yamaguchi, Mami Kayama, Yugo Narita, Norikazu Kawada, Miki Akiyama, Akiko Hagiwara, Yoshimi Suzukamo, Shunichi Fukuhara

**Affiliations:** 1 Department of Adult Nursing/Palliative Care Nursing, School of Health Sciences and Nursing, Graduate School of Medicine, The University of Tokyo, Tokyo, Japan; 2 Department of Psychiatric Nursing, School of Health Sciences and Nursing, Graduate School of Medicine, The University of Tokyo, Tokyo, Japan; 3 Department Psychiatric Nursing, St. Luke's College of Nursing, Tokyo, Japan; 4 Department of Neurology, Graduate School of Medicine, Mie University, Mie, Japan; 5 Department of Neurology, Matsusaka chuo General Hospital, Mie, Japan; 6 Fukuyama Transporting Shibuya Longevity Health Foundation, Hiroshima, Japan; 7 Departmet of Physical Medicine and Rehabilitation, Tohoku University Graduate School of Medicine, Sendai, Japan; 8 Department of Epidemiology and Healthcare Research, School of Public Health, Kyoto University, Kyoto, Japan; 9 iHope International (Institute for Health Outcomes & Process Evaluation Research), Tokyo, Japan

## Abstract

**Background:**

We constructed a concise multidimensional care burden scale that reflects circumstances unique to Japan, with a focus on intractable neurological diseases. We surveyed 646 family caregivers of patients with intractable neurological diseases or stroke using 28 preliminary care burden scale items obtained from qualitative research. The results were used to finalize the feeling of care burden scale (BIC: burden index of caregivers), and verify its reliability and validity.

**Methods:**

The survey was conducted among caregivers providing home health care to patients with intractable neurological diseases (PD [Parkinson's disease], SCD [spinocerebellar degeneration], MSA [multiple system atrophy], and ALS [amyotrophic lateral sclerosis]) or CVA (cerebrovascular accident) using a mailed, self-administered questionnaire between November, 2003 and May, 2004.

**Results:**

Response rates for neurological and CVA caregivers were 50% and 67%, respectively, or 646 in total (PD, 279; SCD, 78; MSA, 39; ALS, 30; and CVA, 220). Item and exploratory factor analyses led to a reduction to 11 items, comprising 10 items from the 5 domains of time-dependent burden, emotional burden, existential burden, physical burden, and service-related burden; and 1 item on total burden. Examination of validity showed a moderate correlation between each domain of the BIC and the SF-8 (Health related quality of life scale, Short Form-8), while the correlation coefficient of the overall BIC and CES-D was 0.62. Correlation between the BIC and ZBI, a preexisting care burden scale, was high (r = 0.84), while that with the time spent on providing care was 0.47. The ICC (Intraclass correlation coefficient) by test-retest reliability was 0.83, and 0.68 to 0.80 by individual domain.

**Conclusion:**

These results show that the BIC, a new care burden scale comprising 11 items, is highly reliable and valid.

## Background

The concept of the burden of care was defined in 1980 by SH Zarit, an American gerontologist, as the discomfort experienced by the principal caregiver of an older family member, including the caregiver's health, psychological well-being, finances, and social life [1]. Since then, a number of burden scales to assess the care burden of family caregivers of impaired or elderly patients have been developed in various countries, including Japan, such as the Zarit Burden Interview (ZBI), Caregiver Strain Index (CSI), Care Burden Inventory (CBI), Caregiver Reaction Assessment (CRA), Care Burden Scale (CBS), and Nakatani's Burden Scale [1-9]. In particular, the ZBI has been validated in Japan and used in a large number of studies [2,10-12]. The ZBI does not, however, have a clear domain structure, nor does it necessarily correspond to various aspects of the care burden [8,13]. In contrast, the CBI and CRA have been developed with a clear domain structure [5-7]. Very few multidimensional scales have been developed in Japan, apart from the CBS developed by Niina, which comprises 28 items covering 9 domains [8]. However, this relatively high number of items is burdensome to respondents.

Japanese health policy now provides various preferential treatment conditions to patients with any of a number of neuromuscular diseases, including amyotrophic lateral sclerosis (ALS), multiple system atrophy (MSA), spinocerebellar degeneration (SCD), and Parkinson's disease (PD), under the framework of "incurable diseases". Despite increased subsidization of costs, however, the heavy burden of home care for these patients has remained [14,15].

Our previous study, Outcomes Research of Specific Diseases (PI: S. Fukuhara), was conducted under the auspices of the Japanese Ministry of Health, Labor and Welfare to investigate the feelings of burden among caregivers of patients, mostly with ALS, and identify those burdens related not only to time constraints and depression, as conventionally noted, but also to the difficulty in finding meaning in prolonging a patient's life and to difficulties in receiving public nursing services [16,17].

Here, we used the results of this qualitative research to construct a concise multidimensional care burden scale that reflects circumstances unique to Japan for caregivers of patients with mainly intractable neurological conditions. Further, we also verified the reliability and validity of the scale.

## Methods

### Participants and procedure

Participants were caregivers providing home healthcare to patients with intractable neurological conditions and to stroke patients between November, 2003 and May, 2004. A self-rating questionnaire was mailed to all caregivers of patients registered as having the intractable neurological conditions PD, SCD, MSA, and ALS in Mie Prefecture, Japan.

The participants were requested to answer the questionnaire and return the answer sheets. As the qualitative interview study progressed, characteristics of the care burden for neuromuscular disease were found to be similar to those for other diseases, including cerebrovascular accident (CVA), which is the leading cause of bedridden elderly in Japan. Therefore, in consideration of the possible application of the instrument to diseases other than intractable neurological conditions, we also included caregivers of patients with CVA. For this CVA component, the questionnaire was sent to outpatient, day care, rehabilitation, and visiting nurse station departments of the Brain Attack Center Oota Memorial Hospital, a neurological hospital in Hiroshima Prefecture, Japan, for distribution to caregivers who provide nursing care at home to patients following cerebral infarction, cerebral hemorrhage, and subarachnoid hemorrhage. These caregivers were similarly asked to answer the questionnaire and return the answer sheets by mail. To examine reliability, two-week test-retesting of the questionnaire was done in consenting caregivers of stroke patients.

### Measurements

Before the survey, a qualitative study was conducted to collect data and interview primary caregivers of patients with PD, ALS, and CVA. A total of 22 caregivers were interviewed and analyzed. This study used the theoretical sampling method. Caregivers were interviewed for 1.5 h at home under written consent with questions such as "what is the hardest thing about caring for the patient?" The data were analyzed using the grounded theory approach and constant comparative method by means of open, axial and selective coding [16,17]. Data were continually analyzed as they were collected, with comparison between newly gathered data and previous data to examine similarities and differences. Analysis was supervised by a qualitative researcher and results were discussed among neurologists and nurses experienced with neurology patients to examine validity.

Characteristic results revealed care burden items such as "difficulty with finding meaning in prolonging life" (anguish of caregivers in continuing care) and difficulty with finding "a sense of satisfaction" due to a lack of appreciation from other family members, relatives, and others. These items had not been included in the conventional scale. In addition, the qualitative study also extracted an item that reflected the current situation of troubled caregivers whose patients resented receiving public nursing care services, despite having agreed to do so before the services were made available. We then combined our qualitative research results and literature review [1-9] to prepare a preliminary group of 28 care burden scale items. A number of items, including overall burden, were adopted from pre-existing care burden scale items and their wording was revised. The 28 care burden items comprised 27 items of specific burdens (e.g. "I do not have enough time for myself," "I am completely distressed by caregiving", et al), assessed using a 5-point Likert scale from 0: never, 1: almost never, 2: sometimes, 3: often, to 4:always; and 1 item of overall burden i.e. "how much burden do you think providing care is to you?" To examine content validity, three caregivers, two neurologists, and two visiting home nurses checked the questionnaire.

The following instruments were also measured to examine construct and concurrent validity:

1. The Health-related QOL Scale SF-8 [18] short form of the health-related QOL scale SF-36. Its 8 items are assessed by a 5- or 6-point Likert scale, and include physical functioning (PF) (5-point Likert scale), role functioning physical (RP) (5), bodily pain (BP) (5), general health perception (GH) (6), vitality (VT) (5), social functioning (SF) (5), role functional emotional (RE) (5), and mental health (MH) (5). In this study, we used each item separately. Higher values represent better QOL.

2. The Center for Epidemiologic Studies Depression scale (CES-D) [19,20], developed by the National Institute of Mental Health, USA, is a self-report scale to identify depression which has been translated into Japanese by Shima. Its 20 symptomatic items are self-assessed to identify the number of days per week a subject is affected by depression, as "none", "1 to 2 days", "3 to 4 days", "5 or more days," providing for a total score of 0 to 60, with a higher score representing a stronger tendency toward depressive feelings.

3. The Zarit Burden Interview [1,2], a care burden scale developed by Zarit and translated into Japanese by Arai, comprises 22 items assessed by a 5-point Likert scale to provide a total score of 0 to 88, with a higher score representing a greater care burden.

Regarding demographic factors, we collected information on caregiver's age, gender, relationship to patient, working status, income, period of nursing, daily hours spent on nursing, hours required for close supervision of the patient, patient's age, level of ADL (as determined by the Ministry of Health, Labor and Welfare), and Barthel Index (for CVA only) [21].

### Statistical analysis

We first prepared a multidimensional scale by selecting items from the 28 preliminary items. In the item-selection process, we performed item analysis to confirm that selected answers were not heavily weighted on either end of the 5-point scale (i.e. 80% or more responders selected points 0 and 1 or 4 and 5), and examined reproducibility with a weighted kappa coefficient to exclude items with κ value < 0.5. On the basis of these results, we confirmed multidimensionality by an explanatory factor analysis (principle method with a promax rotation), applied the item response theory (marginal maximum likelihood method, graded response model) to each factor, and determined the difficulty level and discrimination power of each item. We then adopted those items which included a difficulty level of 0 and a discrimination power of 2 or higher. Finally, we established a 5-dimensional Burden Index of Caregiver (BIC) that contains 11 items.

We then verified the validity and reliability of the BIC. We first calculated the descriptive statistics of scores in each domain, and examined internal consistency using Cronbach's alpha coefficient. We then performed explanatory factor analysis (principle method with a promax rotation) to examine factor validity. To address construct and concurrent validity, we calculated Pearson's correlation coefficient between the BIC and health-related QOL scale (SF-8), depression (CES-D), and preexisting ZBI. To address known groups validity, we summed BIC scores according to the hours spent on caregiving per day, and calculated Spearman's correlation coefficient, r [2,22]. To evaluate reproducibility, we calculated intraclass correlation coefficient (ICC). In addition, we similarly confirmed the unidimensionality of overall care burden feelings, calculated Cronbach's alpha coefficient and ICC to examine the availability of a total score, and calculated correlation of the total score to confirm items of construct and concurrent validity.

We used MULTILOG 7.03 (Assessment Systems Corporation, St. Paul, MN) for item response theory and SAS Version 9.1 (SAS Institute, Cary, NC) for all other analyses.

### Ethical considerations

Before implementing this study, the protocol was reviewed by Ethical Committees at the Faculty of Medicine, the University of Tokyo, Mie University Hospital, and Brain Attack Center Oota Memorial Hospital. Each subject was informed by a written document that participation in the study was voluntary and their privacy would be strictly protected.

## Results

### Subject characteristics

The questionnaire was sent to 1577 patients with intractable neurological diseases and 332 following stroke, and answer sheets were received from 785 (50%) and 220 (67%), respectively. The number of total responders (analysis set) who provided valid final responses was 646 (PD, 279; SCD, 78; MSA, 39; ALS, 30; and CVA, 220).

The average age of caregivers was 64.3 ± 11.6 years, and 64.9% were women (Table 1). The relationship between patient and caregiver was spouse in 72.3%. The employed population was 23%. Among all responders, annual income was less than 3 million yen (US$X) for 41.4% and less than 5 million yen (US$X) for 73.0%. Average duration of caregiving was 5.2 ± 4.4 years, and average time spent on care was 6.0 ± 6.3 hours daily. According to ADL levels determined by the Ministry of Health, Labor and Welfare, 14.3% of patients were Level C (bed rest throughout the day), 34.2% were Level B (need help for daily activity), 34.2% were Level A (independent at home but need some help outside the house), and 17.2% were Level J (almost completely independent) or physically independent.

**Table 1:** Subject characteristics (n = 646)

	N	%
Caregiver's age, y [Mean(SD)]	64.3(11.6)	
Gender (Female)	412	65
Relationship of caregiver		
Spouse	460	71
Child/Child-in-law	138	21
Other	48	7
Caregivers at work	147	23
Household income (Yen, millions)		
<=3	250	41
<=5	191	32
<=7	81	13
<=9	43	7
>9	39	7
Duration of caregiving, y [Mean(SD)]	5.2(4.4)	
Hours spent caregiving per day [Mean(SD)]	6.0 (6.3)	
Hours required for close supervision of the patient [Mean(SD)]	5.2 (6.7)	
Patient's age, y [Mean(SD)]	70.4 (9.6)	
Diagnosis		
PD (Parkinson's disease)	279	43
SCD (spinocerebellar degeneration)	78	12
MSA (multiple system atrophy)	39	6
ALS (amyotrophic lateral sclerosis)	30	5
CVA (cerebrovascular accident)	220	34
Duration of disease, y [Mean(SD)]	8.0 (6.1)	
ADL (Index of activity of daily living by MHLW)		
J/Independent	89	17
A	177	34
B	177	34
C	74	14
SF-8 score [Mean(SD)]		
PF (Physical Functioning)		2.4(1.2)
RP (Role functioning Physical)		2.5(1.2)
BP (Bodily Pain)		3.0(1.3)
GH (General Health perceptions)		3.7(0.9)
VT (Vitality)		2.9(0.9)
SF (Social Functioning)		2.5(1.2)
RE (Role functional Emotional)		2.5 (1.1)
MH (Mental Health)		2.8 (1.1)
CES-D score [Mean(SD)]		15.6 (9.8)
ZBI score [Mean(SD)]		35.0(18.4)
Barthel Index (only CVA) [Mean(SD)]		53.0(34.0)

SF-8: Health related quality of life scale, Short Form-8

CES-D: Center for Epidemiologic Studies Depression scale

ZBI: Zarit Burden Interview

Income: 1 million yen is approximately equivalent to US$8,600

### Item selection and face validity

Care burden items were selected according to the procedures described in Methods. The finalized BIC comprise 11 items, as follows: 1. I do not have enough time for myself because of caregiving, 2. I cannot freely leave the house because of caregiving, 3. I am completely distressed by caregiving, 4. I want to delegate the care to someone else, 5. I am experiencing hardship because caregiving does not give me a sense of satisfaction, 6. Caregiving is hard because I cannot find the meaning of nursing, 7. My body aches when nursing, 8. I have ruined my health because of nursing, 9. I have a hard time because patients resent receiving public nursing care service, 10. It is a burden that public nursing care service personnel enter our house; and regarding the total care burden, 11. All things considered, how much burden do you think providing care is to you?

We assessed the face validity of the survey items among nurses who had experience in caring for patients with either intractable neurological diseases or stroke.

### Factor validity

Five factors were extracted as a result of the exploratory factor analysis performed on the 10 items, excluding the total care burden of the BIC, and named "Time-dependent burden", "Emotional burden", "Existential burden", "Physical burden", and "Service-related burden" (Table 2). Service-related burden demonstrated a slight deviation in distribution and its Cronbach's alpha coefficient was low (alpha = 0.68). For the other domains, however, distributions showed no deviation and internal consistency was high (0.84 to 0.89). The average total score of the BIC was 16.8 (± 8.9) and its Cronbach's alpha coefficient was 0.91.

**Table 2:** Factor analysis, discriptive statistics and internal consistency.

	Factor loading					
	Factor 1	Factor 2	Factor 3	Factor 4	Factor 5	Communality

Time-dependent Burden (0–8: Mean = 4.6, SD = 2.2, alpha = 0.84)						
1. I cannot freely leave the house because of caregiving	0.93	-0.15	0.10	0.07	-0.03	0.89
2. I do not have enough time for myself because of caregiving	0.83	0.19	-0.07	0.01	0.04	0.86
Emotional Burden (0–8: Mean = 2.7, SD = 2.1, alpha = 0.89)						
3. I want to delegate the care to someone else	-0.06	0.91	0.06	0.07	-0.03	0.90
4. I am completely distressed by caregiving	0.07	0.77	0.16	0.05	0.01	0.88
Spiritual Burden (0–8: Mean = 2.8, SD = 2.1, alpha = 0.88)						
5. I am experiencing hardship because caregiving does not give me a sense of satisfaction	0.11	0.04	0.90	-0.01	-0.03	0.91
6. Caregiving is hard because I cannot find the meaning of nursing	-0.06	0.19	0.82	0.00	0.07	0.88
Physical Burden (0–8: Mean = 3.1, SD = 2.3, alpha = 0.86)						
7. My body aches when nursing	0.01	0.05	-0.02	0.92	0.00	0.88
8. I have ruined my health because of nursing	0.10	0.07	0.01	0.82	0.01	0.85
Service-related Burden (0–8: Mean = 1.8, SD = 1.8, alpha = 0.68)						
9. It is a burden that public nursing care service personnel enter our house	0.09	0.22	-0.12	-0.11	0.86	0.80
10. I have a hard time because patients resent receiving public nursing care service	-0.08	-0.20	0.15	0.13	0.85	0.78
11. Total Care Burden (0–4: Mean = 1.9, SD = 1.2)	-	-	-	-	-	-

BIC total (0–44: Mean = 16.8, SD = 8.9, alpha = 0.91)	-	-	-	-	-	-

Total contribution of factors						0.86

Figures indicate standardized regression coefficient

Alpha indicate Cronbach's alpha coefficient

BIC: Burden Index of Caregivers

### Construct and concurrent validity

In terms of construct and concurrent validity, the correlation between BIC and SF-8 was rational as a whole, and was strongest for "Time-dependent burden" and Social Functioning (r = -0.59), "Emotional burden" and "Existential burden" and Mental Health (r = -0.58, -0.55, respectively), and "Physical burden" and Bodily Pain (r = -0.64) (Table 3). Correlation was weak between "Service-related burden" and the SF-8. The correlation coefficient between the BIC total score and each SF-8 item ranged from -0.47 to -0.65. In contrast, that between each domain in the BIC and CES-D ranged from0.32 to 0.54; that of the BIC total score and CES-D was 0.62; that between each domain in the BIC and ZBI ranged from 0.38 to 0.76; and that for BIC total score and ZBI was high, at 0.84.

**Table 3:** Construct and concurrent validity

	Time-dependent Burden	Emotional Burden	Exisitential Burden	Physical Burden	Service-related Burden	Total Care Burden	BIC Total
SF-8							
PF (Physical Functioning)	-0.36	-0.35	-0.40	-0.52	-0.26	-0.44	-0.52
p-value	0.0001	0.0001	0.0001	0.0001	0.0001	0.0001	0.0001
RP (Role functioning Physical)	-0.47	-0.48	-0.49	-0.57	-0.28	-0.56	-0.65
p-value	0.0001	0.0001	0.0001	0.0001	0.0001	0.0001	0.0001
BP (Bodily Pain)	-0.41	-0.38	-0.40	-0.64	-0.26	-0.44	-0.56
p-value	0.0001	0.0001	0.0001	0.0001	0.0001	0.0001	0.0001
GH (General Health perceptions)	-0.39	-0.40	-0.40	-0.56	-0.27	-0.46	-0.59
p-value	0.0001	0.0001	0.0001	0.0001	0.0001	0.0001	0.0001
VT (Vitality)	-0.35	-0.41	-0.41	-0.50	-0.26	-0.41	-0.47
p-value	0.0001	0.0001	0.0001	0.0001	0.0001	0.0001	0.0001
SF (Social Functioning)	-0.59	-0.48	-0.49	-0.54	-0.31	-0.57	-0.62
p-value	0.0001	0.0001	0.0001	0.0001	0.0001	0.0001	0.0001
RE (Role functional Emotional)	-0.52	-0.50	-0.51	-0.55	-0.25	-0.58	-0.60
p-value	0.0001	0.0001	0.0001	0.0001	0.0001	0.0001	0.0001
MH (Mental Health)	-0.55	-0.58	-0.55	-0.55	-0.29	-0.59	-0.64
p-value	0.0001	0.0001	0.0001	0.0001	0.0001	0.0001	0.0001
CES-D	0.49	0.54	0.53	0.48	0.32	0.51	0.62
p-value	0.0001	0.0001	0.0001	0.0001	0.0001	0.0001	0.0001
ZBI	0.76	0.72	0.70	0.68	0.38	0.80	0.84
p-value	0.0001	0.0001	0.0001	0.0001	0.0001	0.0001	0.0001

figures indicate Pearson's correlation coefficient

BIC: Burden Index of Caregivers

SF-8: Health related quality of life scale, Short Form-8

CES-D: Center for Epidemiologic Studies Depression scale

ZBI: Zarit Burden Interview

### Known groups validity

**Figure 1:**
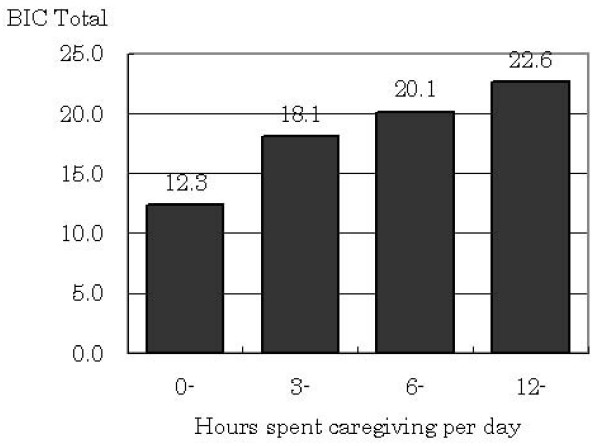
Know groups validity

Known groups validity was examined using the correlation between BIC total score and actual hours spent on daily caregiving (Fig. 1). The correlation between actual caregiving hours and a feeling of care burden has been previously raised in other studies [2,22]. On average, the BIC total score was 12.3 for less than 3 hours, 18.1 for 3 to less than 6 hours, 20.1 for 6 to less than 12 hours, and 22.6 for 12 hours or more. Spearman's ρ was 0.47, and indicated a statistically significant correlation (*p *< 0.001).

### Reliability

ICC, an indicator of reliability, was 0.77 for "Time-dependent burden", 0.73 for "Emotional burden", 0.70 for "Existential burden", 0.80 for "Physical burden", 0.68 for "Service-related burden", and 0.70 for "Total care burden" (Table 4). The ICC was 0.83 for the BIC total score, indicating sufficient reliability.

**Table 4:** Reliablity

	ICC	95% Conficence Interval		
		lower		upper
Time-dependent Burden	0.77	0.69	-	0.83
Emotonal Burden	0.71	0.60	-	0.78
Exsitential Burden	0.73	0.64	-	0.80
Physical Burden	0.80	0.73	-	0.85
Service-related Burden	0.68	0.58	-	0.76
Total Care Burden	0.71	0.60	-	0.78
BIC Total	0.83	0.76	-	0.87

ICC: Intraclass Correlation Coefficient

BIC: Burden Index of Caregivers

## Discussion

Given the paucity of multidimensional care burden scales in Japan, the present findings confirming the reliability and validity of the BIC, a short multidimensional scale which measures 5 domains with 11 items, are significant. Moreover, the BIC adds two new domains, existential burden and service-related burden, developed on the basis of qualitative research. The merit of the BIC multidimensional scale is that it allows the evaluation of responsiveness for intervention. Generally, intervention trials focus on some aspect of care burden. The multidimensional BIC scale not only yields high responsiveness for the targeted area, but also provides detailed information on the effectiveness of an intervention. To date, Japan has had no suitable multidimensional scale with this aim. In addition, the new subscale "service-related burden" is expected to contribute to evaluation of the new Japanese nursing insurance system. This BIC, which is not a translation of an instrument developed outside Japan, meets the needs of those who have sought a scale that reflects the particular circumstances of this country.

In addition to the CBS [8], which comprises 28 items in 9 domains, several other 3-dimensional scales have been developed as multidimentional scales in Japan. However, these scales have different domains from the BIC – Higashino [23], for example, includes economical issues as one domain, while Hashimoto [13] dealt with satisfaction with caregiving as a positive feeling. The BIC somewhat resembles the CBI and CRA, although the composition of their domains is not identical [5-7].

The correlation between the BIC and health-related QOL scale SF-8 shows that the domains of the BIC most strongly correlated with different items of the SF-8. For the SF-8, we assumed that PF would correlate with physical burden before the study began. However, results showed that physical burden was more strongly correlated with bodily pain (r = -0.64) than PF (r = -0.52). We interpret this result to mean that caregivers' physical burden affected physical functioning through the bodily pain arising from daily caregiving. This finding suggests that the BIC can be used in domain-based assessments of outcome of interventions conducted to alleviate feelings of care burden and to provide highly sensitive endpoints that align with the focus of interventions. The BIC total score is highly correlated with that of the CES-D, indicating that results obtained with the BIC will tend to be consistent with those of previous studies [24,25].

The items used in this study were derived primarily from qualitative investigation of intractable neurological diseases and reviews of past studies. We were therefore able to extract the previously unidentified domain of existential burden, which includes "a sense of satisfaction" and "cannot find the meaning of caregiving". This domain may be linked to the characteristics of intractable neurological conditions, namely that they are progressive and that their progression is unpredictable [26-28]. These present results suggest that, in addition to conventional interventions that reduce time and physical burden such as home-help services and nursing day care, minimization of the burden of care requires an understanding of what caregivers think about nursing care, and the provision of support for the existential aspects of care.

Further, we extracted nursing service-related burden as a new domain in this study. A recent study by Hashimoto [13] incorporated a similar burden into the concept of "a sense of isolation," but not as an independent domain. This concept may be distinctive of or unique to Japanese care settings, and may be linked to the recent institutionalization of the public nursing care insurance system, which has expanded as a surrogate for care hitherto provided by family members. The BIC is therefore considered suitable for assessing the effectiveness and acceptance of nursing services provided by the government.

In this paper, we have demonstrated the validity and reliability of the BIC, a new multidimensional scale. Progression and impairment differ among patients with PD, MSA, SCD, ALS, and CVA, the diseases targeted in this study, and feelings regarding the burden of care will be influenced by the type of disease. As a next step, our task is to investigate factors related to the feeling of care burden as classified by disease, and identify suitable plans for intervention.

The limitations of this study are as follows. First, the response rate among study participants with intractable neurological diseases was low (50%). We suspect that this is related to the patient register used, which included a considerable number of people who do not require care. Thus, the true response rate might be greater than the nominal value. However, it is a fact that there is a lack of external validity in this study. Unfortunately, we were unable to compare background factors between responders and non-responders owing to a law protecting personal information in Japan and our use of a sample registered under a local governmental registration system for intractable disease. Second, the subjects were restricted to caregivers providing nursing care to patients with intractable neurological diseases or CVA at home. Application to other diseases thus requires further research. Third, this study examined reliability and validity through a cross-sectional study only, and thus requires further evaluation of responsiveness before and after intervention and in longitudinal studies.

## Conclusion

We constructed the BIC, a concise multidimensional care burden scale which comprises 11 items covering 5 domains, from qualitative study of 646 family caregivers who provide care at home to patients with intractable neurological diseases or stroke. Further, we verified that the BIC has sufficient reliability and validity.

## Competing interests

The author(s) declare that they have no competing interests.

## Authors' contributions

MM was involved in design, collecting data, analysis of data, interpretation, and drafting manuscript.

YA was involved in design, collecting data and analysis of data.

KM was involved in design, interpretation, and revising the paper for important intellectual content.

NY was involved in design and collecting data.

KN was involved in design and collecting data.

AM was involved in collecting data, analysis of data.

HA was involved in collecting data.

SY was involved in design, interpretation, and revising the paper for important intellectual content.

FS was involved in design, interpretation, and revising the paper for important intellectual content.

## Appendix

**Figure 2:**
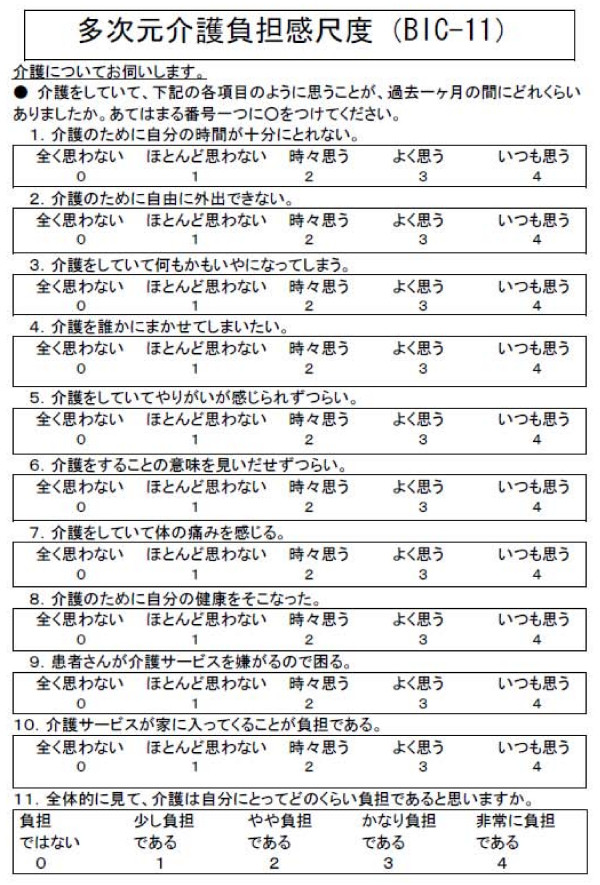
Burden Index of caregivers (BIC)

Figure 2 shows the Japanese wording of the BIC scale. The BIC is managed by iHope International (Institute for Health Outcomes & Process Evaluation Research), and the scale and instruction manual can be downloaded from the iHope website [29]
